# The Early Variation of Left Ventricular Strain after Aortic Valve Replacement by Three-Dimensional Echocardiography

**DOI:** 10.1371/journal.pone.0140469

**Published:** 2015-10-16

**Authors:** Yongle Chen, Zhiqi Zhang, Leilei Cheng, Li Fan, Chunsheng Wang, Xianhong Shu

**Affiliations:** 1 Shanghai Institute of Medical Imaging, Department of Echocardiography, Zhongshan Hospital, Fudan University, Shanghai Institute of Cardiovascular Diseases, Shanghai, P.R.China; 2 Department of Cardiac Surgery, Zhongshan Hospital, Fudan University, Shanghai Institute of Cardiovascular Diseases, Shanghai, P.R.China; Georgia Regents University, UNITED STATES

## Abstract

Aortic stenosis (AS) and aortic incompetence (AI) are common aortic valve diseases. Both may deteriorate into irreversible myocardial dysfunction and will increase the risk of sudden death. In this study, we aimed to investigate the early variation trend of left ventricular function by three-dimensional speckle tracking echocardiography (3D-STE) in the patients who underwent cardiac surgeries for aortic valve disease. Twenty patients with severe aortic AS and 16 patients with severe AI were enrolled. All of them underwent the aortic valve replacement (AVR) procedures. The patients’ global longitudinal strain (GLS) and global circumferential strain (GCS) were evaluated by 3D-STE before surgery and at 1 week after surgery. In addition, GLS and GCS were followed at 1 month as well as 3 months after AVR. In AS patients, the GCS after AVR altered little both at 1 week (*p = 0*.*562*) and at 1 month (*p = 0*.*953*) compared with the data before the surgery. And it increased significantly at 3 months of follow-up observation compared to that before AVR (*p<0*.*05*). Meanwhile, GLS increased progressively after AVR and improved significantly at 3 months after surgery (*p<0*.*05*). For the AI patients, GLS as well as GCS decreased at 1 week after AVR compared to those data at baseline (*p<0*.*05*). However, these two parameters recovered at 1 month after AVR. Furthermore, GLS and GCS improved significantly at 3 months after the surgery (*p<0*.*05*). Therefore, both GLS and GCS were influenced by AVR and would be improved at 3 months after surgery both in AS patients or AI patients. GLS and GCS can be finely evaluated by 3D-STE, and they are helpful to determine the variation tendency of left ventricular function in patients with AVR.

## Introduction

Both aortic stenosis (AS) and aortic incompetence (AI) are common aortic valve diseases. Severe aortic stenosis is often associated with concentric hypertrophy of left ventricle (LV) which is caused by a high afterload. On the other hand, severe aortic incompetence mainly induces the increasing of LV volume. More than one-third of patients with severe AS and more than one-fourth with AI showed symptoms [1–3]. Those patients usually suffer from the symptoms of left heart failure like fragile, dyspnea, chest congestion or orthopnea. Both severe AS and severe AI patients will deteriorate into irreversible myocardial dysfunction such as left ventricular heart failure which may increase the risk of sudden death. Aortic valve replacement (AVR) is an effective therapy which may reduce the potential risk of sudden death and improve left ventricular heart function as well [4–5]. However, the predicting factors for the timing of surgery are still being discussed. Besides, the mechanism of the improvement on the left ventricular function remains unclear.

Since the deteriorated left ventricular (LV) function caused by severe AI or severe AS is derived from myocardium, the parameters on the detection of LV contraction may be related with the LV conditions of AI or AS patients both before and after surgery. As we all know, LVEDV and LVESV stand for primary evaluation of left ventricular function. Compared with left ventricular ejection fraction (LVEF), diameters of left ventricle and thickness of left ventricular wall, the strain imaging in echocardiography may distinguish the passive contraction from active contraction of LV. This technique is widely used in directing the therapy and in predicting the prognosis of patients with cardiovascular diseases [6–10]. Nowadays, more and more studies have been concentrated on left ventricular strain in AS and AI patients by 2-dimensional (2D) speckle tracking imaging. But the results of those studies were numerous and uneven [11–14].

Three-dimensional (3D) echocardiography has been thrived in recent years for its superiorities of rapid image acquisition and monolithic image reconstruction. Three-dimensional echocardiography can obtain the whole information of cardiac ventricle and provide a more visualized image than two-dimensional echocardiography. Thus, it is widely used in evaluating valvular diseases, left ventricular dyssynchrony, regional function, pericardial diseases and congenital heart diseases [15–17].

The purposes of this study is to investigate the variation of left ventricular function by the novel 3D speckle tracking echocardiography for severe AS or severe AI patients who have underwent aortic valve replacement. In addition, we demonstrate the application value of 3D speckle tracking imaging.

## Material and Methods

### Study Population

The study was approved by the ethical committee of Zhongshan Hospital (Approval number: B2013-105) and the informed consent was signed by every patients.

Thirty-six consecutive patients who underwent AVR by same surgeon in Zhongshan Hospital, Fudan University from April to October 2013 were enrolled. Including 20 patients with severe AS (Aortic Vmax ≥ 4m/s or AVA ≤1.0cm^2^) and 16 patients with severe aortic valve incompetence [18]. The patients were excluded if they had a history or presented with: (1) one or more coronary artery need percutaneous coronary intervention; (2) previous myocardial infarction; (3) concomitant coronary artery bridging graft surgery (CABG); (4) more than mild additional valve diseases.

### Echocardiography protocol

The commercially available echocardiographic systems (Philips IE 33, Philips Medical Systems Corporation, MA, USA) was applied, equipped with S5-1 (1–5 MHz) and X3-1 (1–3 MHz) transducers. All echocardiographic examinations were conducted by two experienced cardiologists. The patients received conventional echocardiography, two-dimensional and three-dimensional echocardiography before the surgery as well as at 1 week, 1 month and 3 months after AVR. The digital data were stored in DICOM format.

All the images were analyzed using Tomtec Imaging System (4D LV-Analysis 3.1, Tomtec Corporation, Germany). Offline measurements were processed by a qualified observer. LVEF, left ventricular end diastolic volume (LVEDV), left ventricular end systolic volume (LVESV) and stroke volume (SV) were derived from 3D volume rendering. Meanwhile, global longitudinal strain (GLS) and global circumferential strain (GCS) were evaluated by 3D speckle tracking echocardiography.

Furthermore, twenty images were selected randomly and reanalyzed by the same observer and another observer for the assessment of intra-observer and inter-observer variability.

### Statistical Analysis

All results were summarized as mean ± SD. The independent Student’s *t* test was used for comparing results of AS and AI group. The paired Student’s *t* test was used for comparing changes within AS and AI patients. Results were considered statistically significant for two-sided *p*< 0.05. All data were analyzed using SPSS 16.0 software (SPSS Inc., Chicago, IL, USA).

## Results

### The baseline characteristics

The clinical characteristics of 20 severe AS patients and 16 severe AI patients before AVR were shown on Table 1. The LVEDV and LVESV of AI patients were much larger than those in AS patients. There was occurrence of prosthesis-patient mismatch after surgery (Table A in S1 File) (Table A in S2 File).

**Table 1:** Clinical characteristics of AS patients and AI patients.

	AS patients (n = 20)	AI patients (n = 16)
Men (n, %)	10 (50%)	10 (62.5%)
Age(years)	56.3±13.9	55.2±14.3
LVEDV (ml)	100.1±15.1	170.4±44.6
LVESV(ml)	54.9±16.0	100.6±35.5
LVEF (%)	45.9±9.2	42.1±9.8
GLS (%)	-11.4±4.2	-13.1±4.5
GCS (%)	-17.2±7.4	-14.5±5.3

AS = aortic stenosis; AI = aortic incompetence; LVEDV = left ventricular end diastolic volume; LVESV = left ventricular end systolic volume; LVEF = left ventricular ejection fraction; GLS = global longitudinal strain; GCS = global circumferential strain.

### The changes of 3D speckle tracking imaging after AVR on AS patients

**Fig 1:**
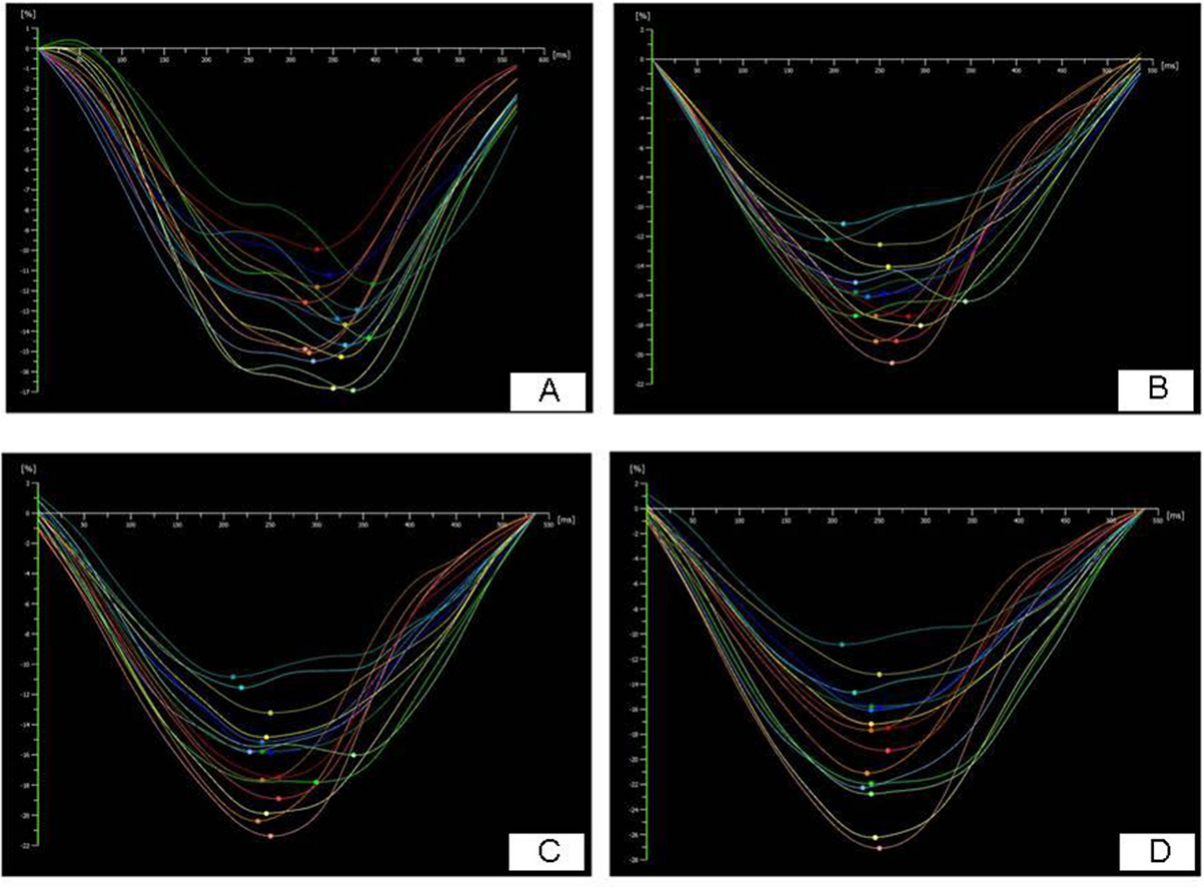
A demonstration of the variety of longitudinal strain of AS patients at baseline, 1 week, 1 month and 3 months after AVR. (A) baseline, GLS = -13.6%; (B) 1 week after AVR, GLS = -15.8%; (C) 1 month after AVR, GLS = -16.3%; (D) 3 months after AVR, GLS = -19.0%).

**Fig 2:**
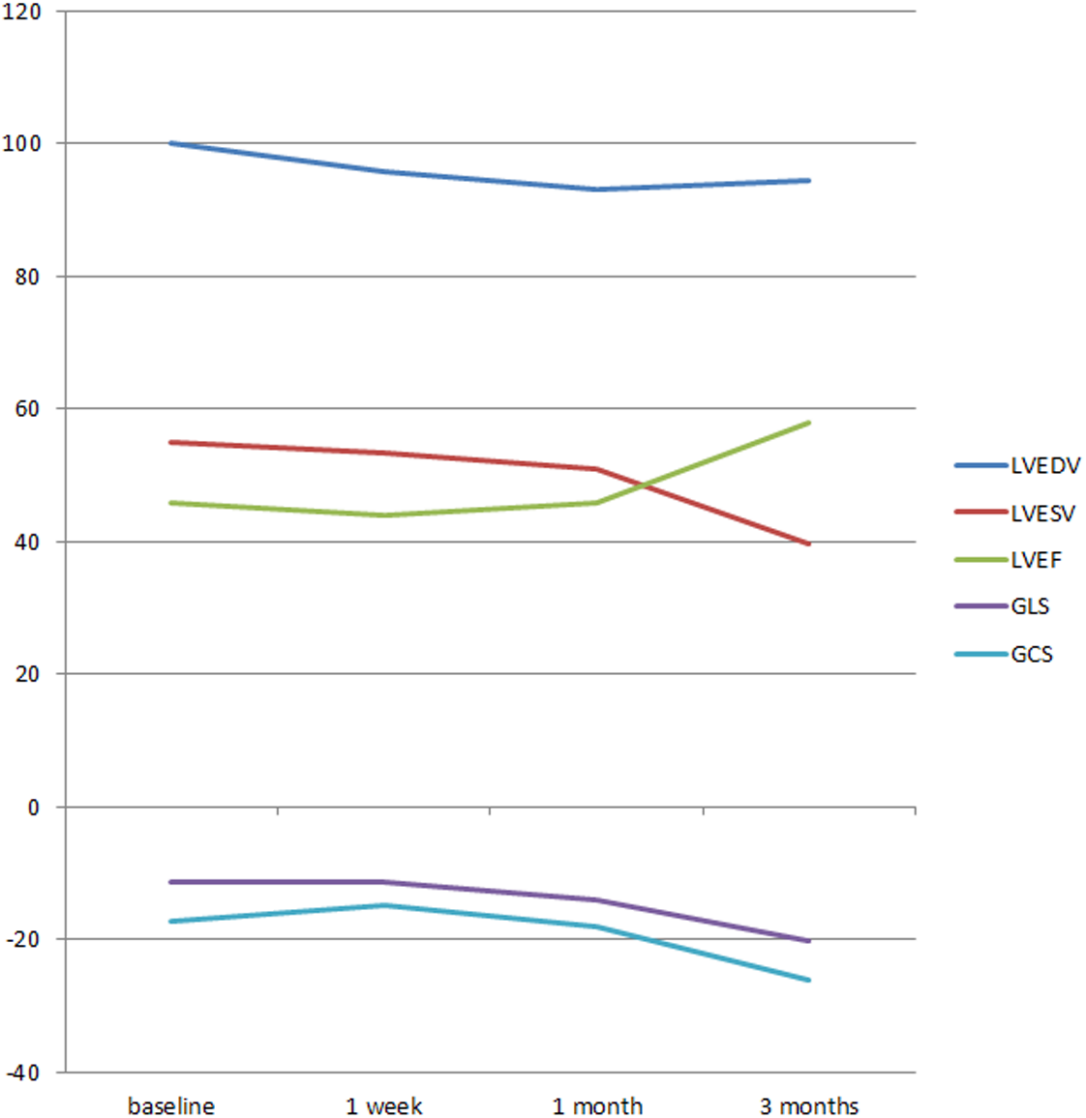
A illustration of the variety of LVEDV, LVESV, LVEF, GLS and GCS of AS patients at baseline, 1 week after AVR, 1 month after AVR and 3 months after AVR.

GCS in AS patients after AVR decreased at 1 week compared to that before surgery. But it did not achieve the statistical significance (-15.0% vs. -17.2%, *p = 0*.*562*) (Table B in S1 File). Afterwards, GCS recovered at 1 month compared to that before surgery (-18.0% vs. -17.2%, *p = 0*.*953*) (Table C in S1 File). After 3 months follow-up, it increased significantly when comparing to that before the cardiac surgery (-26.1% vs. -17.2%, *p<0*.*05*) (Table D in S1 File). In contrast, GLS in AS patients increased progressively after AVR. It improved obviously at 3 months after the intervention (-20.2% vs. -11.4%, *p<0*.*05*). (Table 2, Figs 1 and 2)

**Table 2:** Characteristics before and after surgery in AS patients.

	Before AVR	1 week after AVR	1 month after AVR	3 months after AVR
LVEDV (ml)	100.1±15.1	95.7±12.3	93.1±10.8	94.3±10.0
LVESV (ml)	54.9±16.0	53.4±14.0	50.8±12.6	39.5±7.0
LVEF (%)	45.9±9.2	43.9±8.6	45.8±8.3	58.0±4.8*
GLS (%)	-11.4±4.2	-11.5±5.1	-14.0±5.6	-20.2±4.7*
GCS (%)	-17.2±7.4	-15.0±6.8	-18.0±8.3	-26.1±6.3*

*p<0.05, before versus after aortic valve replacement surgery

AVR = aortic valve replacement; LVEDV = left ventricular end diastolic volume; LVESV = left ventricular end systolic volume; LVEF = left ventricular ejection fraction; GLS = global longitudinal strain; GCS = global circumferential strain; AVR = aortic valve replacement.

### The changes of 3D speckle tracking imaging after AVR on AI patients

**Fig 3:**
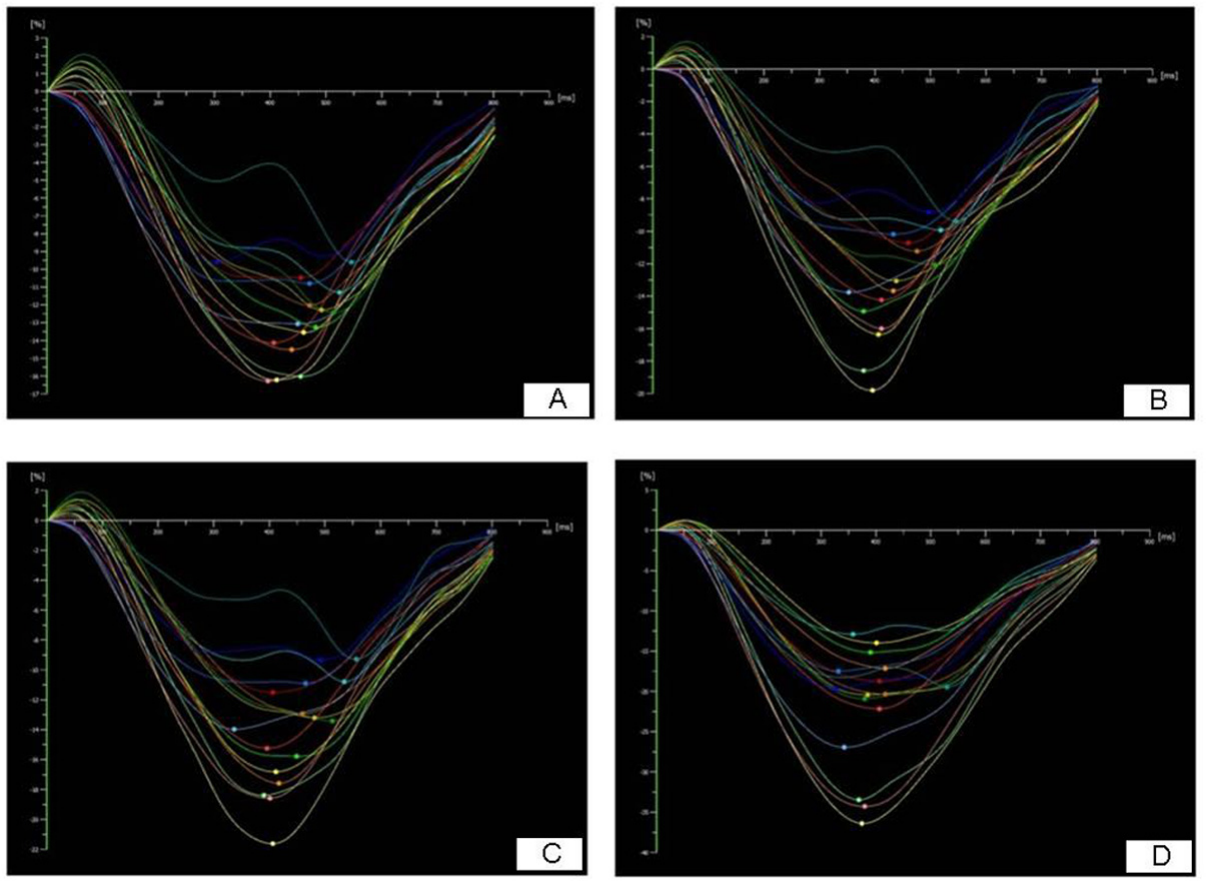
A demonstration of the variety of longitudinal strain of AI patients at baseline, 1 week, 1 month and 3 months after AVR. (A) baseline, GLS = -12.5%; (B) 1 week after AVR, GLS = -13.0%; (C) 1 month after AVR, GLS = -14.1%; (D) 3 months after AVR, GLS = -22.2%).

**Fig 4:**
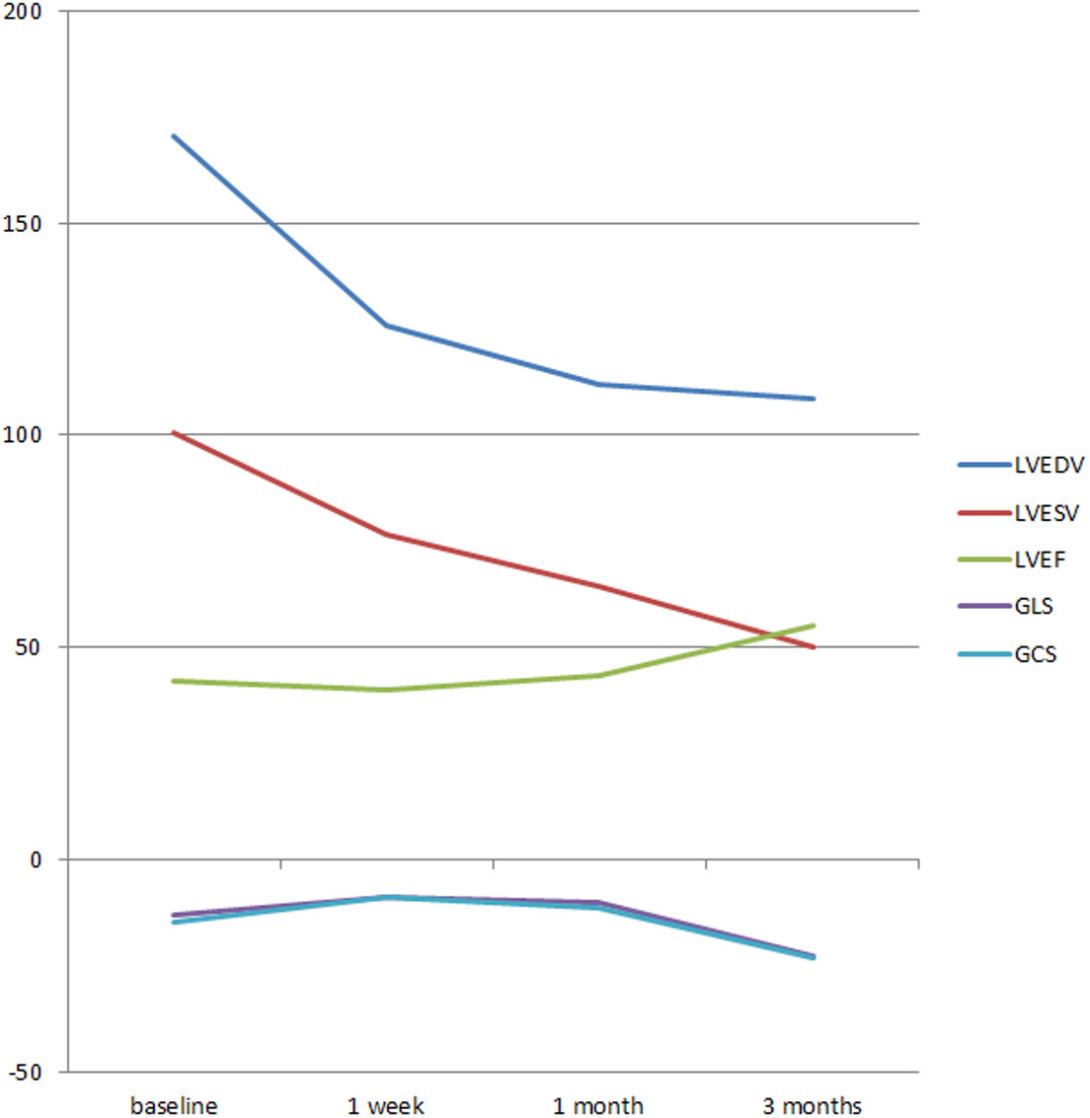
A illustration of the variety of LVEDV, LVESV, LVEF, GLS and GCS of AI patients at baseline, 1 week after AVR, 1 month after AVR and 3 months after AVR.

There were significant decrease of GLS and GCS at 1 week after AVR compared to that before AVR (GLS: -9.0% vs. -13.1%, *p<0*.*05*; GCS: -8.8% vs. -14.5%, *p<0*.*05*) (Table B in S2 File). Both GLS and GCS recovered slowly at 1 month after AVR (GLS: -10.1% vs. -13.1%, *p = 0*.*14*; GCS: -11.4% vs. -14.5%, *p = 0*.*16*) (Table C in S2 File). Consequently, significantly improvements of GLS and GCS were observed at 3 months after AVR (GLS: -22.7% vs. -13.1%, *p<0*.*05*; GCS: -23.3% vs. -14.5%, *p<0*.*05*). (Table 3, Figs 3 and 4) (Table D in S2 File)

**Table 3:** Characteristics before and after surgery in AI patients.

	Before AVR	1 week after AVR	1 month after AVR	3 months after AVR
LVEDV (ml)	170.4±44.6	126.0±35.7	111.8±26.9	108.6±22.3
LVESV (ml)	100.6±35.5	76.4±26.3	64.6±24.5	49.9±17.0
LVEF (%)	42.1±9.8	39.9±9.2	43.4±12.8	55.2±8.0 *
GLS (%)	-13.1±4.5	-9.0±4.0 *	-10.1±4.3	-22.7±4.6 *
GCS (%)	-14.5±5.3	-8.8±4.8 *	-11.4±4.1	-23.3±4.9 *

*p<0.05, before versus after aortic valve replacement surgery

AVR = aortic valve replacement; LVEDV = left ventricular end diastolic volume; LVESV = left ventricular end systolic volume; LVEF = left ventricular ejection fraction; GLS = global longitudinal strain; GCS = global circumferential strain; AVR = aortic valve replacement.

### Inter and intra-observer variation

**Fig 5:**
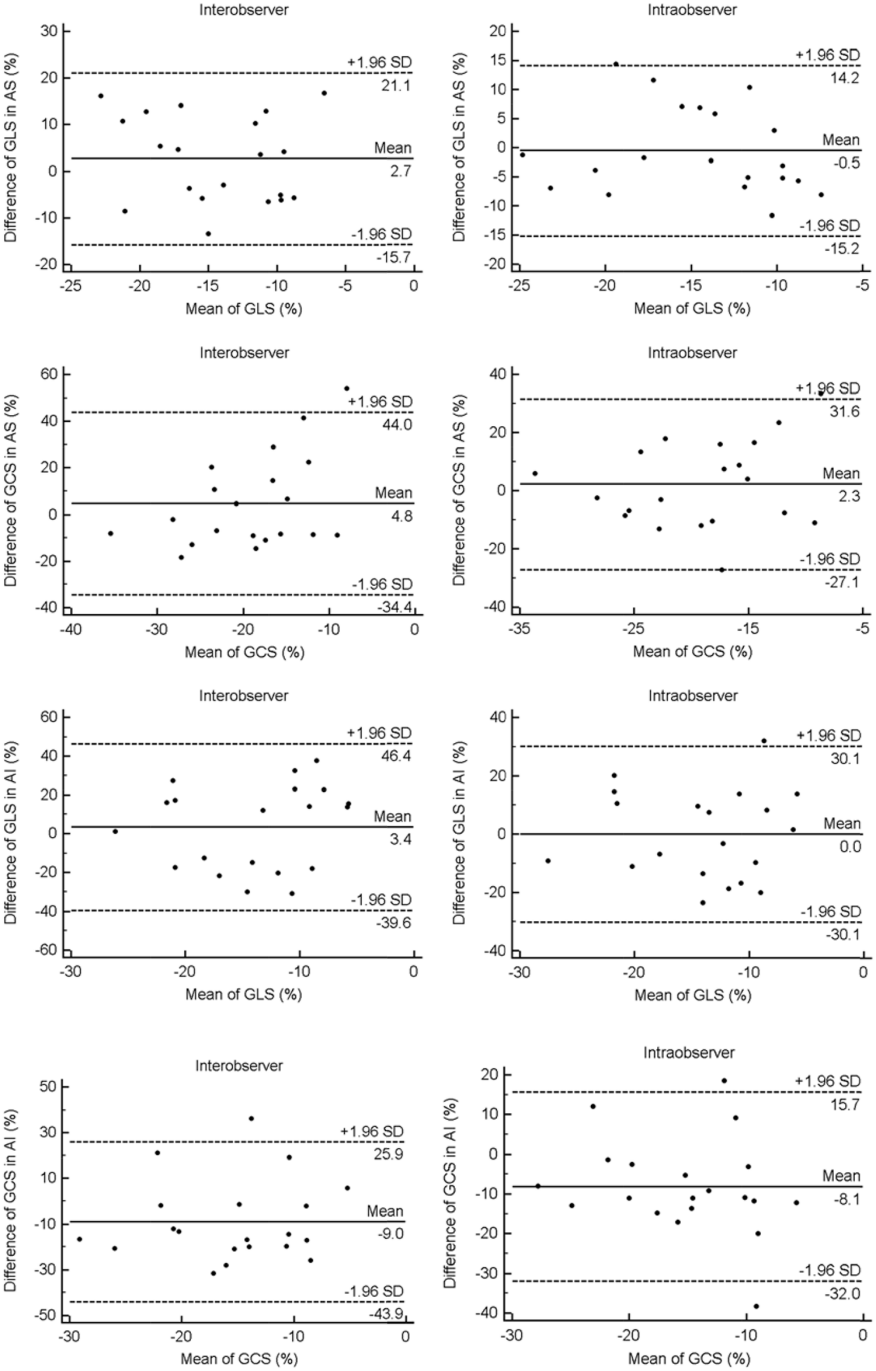
Bland-Altman plots showed inter and intra-observer differences of GLS and GCS in AS and AI patients.

The intraclass correlation coefficient (ICC) was calculated to evaluate the reliability of inter and intra-observer variation. For inter-observer, the ICC of GLS and GCS in AS and AI patients were 0.95, 0.91, 0.88 and 0.89. For intra-observer, the ICC of GLS and GCS in AS and AI patients were 0.97, 0.93, 0.94 and 0.96. The results which were displayed in figure indicated satisfying reproducibility by Bland-Altman analysis (Fig 5).

## Discussion

The conventional 2D speckle tracking imaging (STI) was wildly used in AS and AI patients before and after AVR in order to evaluated the mechanical abnormality and the influences of surgeries. But the results of different studies varied [6–10]. On the other hand, 3D echocardiography is increasingly applied for clinical practice. 3D speckle tracking showed impressively high sensitivity and specificity in evaluating left ventricular function [19], while the intra- and inter-observer agreement for 3D-STI is quite low. However, there were limited studies published, in which 3D echocardiography was involved to evaluate the global strain of LV in AS or AI patients. In this study, we enrolled 20 severe AS patients and 16 severe AI patients who had undergone AVR. By means of 3D speckle tracking echocardiography, we demonstrated the trends of left ventricular global longitudinal strain and global circumferential strain after aortic valve replacement which could be the predictors for LV function. For comparison, we also analyzed LVEF before and after the procedure.

In severe AS and AI patients, the left ventricular systolic parameter LVEF were obviously impaired before surgery. For those AS patients, severe aortic stenosis plays an important role in the obstruction of left ventricle outflow tract (LVOT). The increase of LV afterload contributes to the rise of both systolic pressure and diastolic pressure. Due to the obstruction of LVOT, the left ventricular ejection time is prolonged. Thus, the diastolic time of LV is relatively shortened. Then the time of coronary artery fulfilling is decreased, which induces less oxygen supply. Consequently, the high afterload pressure and the short diastolic time reduce the flexibility of LV, which leads to the weak movement of myocardium. The impaired global longitudinal or circumferential strain can be measured by echocardiography.

The pathophysiological changes of LV in AI patients are much different to AS patients. The severe aortic regurgitation causes overfilling of left ventricle and directly enlarges the volume of LV. The large volume raises the systolic pressure of LV and demands more work of myocardium. As the systolic work increases, the myocardium consumes more oxygen. Furthermore, the larger the volume is, the longer ejection period LV requires. Thus, the compensation of LV function is fragile like walking on ice. Once it is broken up, LVEDV and LVESV will be increased rapidly and LV function will deteriorate definitely as a result. As a result in our study, LVEDV and LVESV showed enlarged, while GLS and GCS showed decreased in AI patients.

In order to break up the cascade of deterioration of left ventricular function, the aortic valve replacement would be a solution. It is expected that AVR could improve LV function and might reduce the enlarged LVEDV and LVESV. As showed in the results, LVEDV and LVESV were both reduced significantly after AVR in both AI and AS patients. But, LVEF, GLS and GCS are not satisfying at one week after surgery. This result is similar with some other reports. Carasso et al. reported that early post AVR (7±3 days) of AS patients, LV size and LVEF did not change. Furthermore, the circumferential strain of mid LV decreased early post AVR [12]. As their study was already showed the results using 2D echocardiography, we performed the similar experiment with 3D echocardiography. The decreases of LVEF, GLS and GCS were observed in our study. AS 3D echocardiography can provide the entire movements of left ventricle, it has the superiority in image reconstruction. We also found that at 1 week after AVR, the decreasing can be observed not only in severe AS patients, but also in severe AI ones.

When followed up to 3 months after AVR, the LVEF, GLS and GCS in AS or AI patients shows an inspiring improvement. These results happened to coincide with the other study, in which the results were obtained from 2D echocardiography [20]. In this study, Rost et al analyzed 40 patients after AVR, and found out myocardial function significantly recovered after the surgery. Another study declared global peak longitudinal strain was reduced for at least 30 days after AVR [21]. The populations in that study including the patients undergoing coronary artery bypass grafting, AVR or combination of other procedures. So these results already showed the 2D-STE could help evaluating left ventricular function, but might not be accurate enough. In our study, we followed the isolated AVR patients up for 3 months. At the moment of 1 month after surgery, LVEF, GLS and GCS evaluated by 3D-STE did not improved significantly compared to those before surgery. But they were obviously elevated, compared to those at one week after surgery. Throughout the whole following period, LVEF, GLS and GCS improved progressively after surgery.

At the present day, both the pathophysiological changes after AVR and the prognosis of post-AVR patients were widely discussed. The timing for aortic valve surgery is the ultimate concern of cardiac surgeons. More and more studies were performed to figure out the situation. Thus, the AVR might reverse the compensatory mechanics in AI patients was reported [22]. But, the mechanism of the variants of strain before and after AVR was still not identified. The latent impact factors include microvessel perfusion of myocardium, fibrosis degree of left ventricular myocardium, surgery and atrial fibrillation. The prognostic value of strain was investigated as well. Low GLS before surgery might be a prognostic factor for AI patients undergoing AVR was reported [14]. GLS might be used in determining the timing of aortic valve surgery was published [3]. In recent ESC/EACTS and AHA/ACC guideline, the echocardiographic diagnosis set the golden standard and several new indicators were introduced. Therefore, our study provided a description of variation trend in left ventricular function both before and after AVR by means of 3D speckle tracking echocardiography. GLS and GCS were sensitive in evaluating the left ventricular function. They might be helpful in discussion of surgery and further studies on pathophysiology and prognosis.

There were some limitations in our study. The number of patients was limited, because we enrolled the patients suffering isolated aortic valve disease in a short period of time. Another limited condition was that the surgical procedures were performed by exact same group of surgeons. As most patients who came to our center had already suffered from cardiac failure for a long time, the patients of both groups had conspicuous low LVEF. Further studies and more patients will be necessary to establish clinical cut off value of the strain parameters indicating the timing for intervention. 3D speckle tracking echocardiographic measurements are, like all echocardiographic techniques, dependent on the quality of image. The endocardial border tracing might be modified by manual in order to improve the track. No histological examinations were performed in our study. Furthermore, we followed up patients for up to 3 months after surgery and the myocardium remodeling might need long term observation.

## Conclusions

GLS and GCS were influenced by AVR and would recover in 1 month in AS patients, while the recovery was slower in AI patients after AVR. GLS and GCS would improve at 3 months after AVR in both AS patients and AI patients. As a result, GLS and GCS could be finely evaluated by 3D speckle tracking echocardiography. 3D-STE showed an accurate evaluation of left ventricular function before and after the valve replacement surgery.

## Supporting Information

S1 File The detail information of echocardiographic parameters of AS patients. (DOCX)

S2 File The detail information of echocardiographic parameters of AI patients. (DOCX)
