# Supplementary material for: The Early Variation of Left Ventricular Strain after Aortic Valve Replacement by Three-Dimensional Echocardiography
Source: PLoS One. 2015 Oct 16;10(10):e0140469. doi: 10.1371/journal.pone.0140469 (PMC4608801; doi:10.1371/journal.pone.0140469)
Supplement: S1 File — (DOCX) [file pone.0140469.s001.docx]

Table A The detail information of echocardiographic parameters of AS patients before AVR

| No. | LVEDV(ml) | LVESV(ml) | LVEF(%) | GLS(%) | GCS(%) |
| --- | --- | --- | --- | --- | --- |
| 1 | 96 | 50 | 48 | -16 | -21 |
| 2 | 110 | 65 | 41 | -8 | -13 |
| 3 | 92 | 49 | 46 | -9 | -16 |
| 4 | 92 | 40 | 56 | -13 | -19 |
| 5 | 82 | 35 | 57 | -16 | -29 |
| 6 | 120 | 79 | 34 | -6 | -10 |
| 7 | 100 | 59 | 41 | -10 | -17 |
| 8 | 83 | 36 | 56 | -16 | -27 |
| 9 | 95 | 50 | 47 | -13 | -19 |
| 10 | 117 | 81 | 31 | -7 | -12 |
| 11 | 121 | 73 | 40 | -5 | -8 |
| 12 | 83 | 47 | 43 | -14 | -25 |
| 13 | 106 | 68 | 36 | -7 | -10 |
| 14 | 81 | 32 | 60 | -15 | -21 |
| 15 | 85 | 34 | 60 | -14 | -24 |
| 16 | 101 | 66 | 35 | -5 | -7 |
| 17 | 97 | 58 | 40 | -13 | -18 |
| 18 | 90 | 37 | 58 | -20 | -28 |
| 19 | 128 | 69 | 46 | -10 | -9 |
| 20 | 123 | 71 | 42 | -10 | -8 |

Table B The detail information of echocardiographic parameters of AS patients at 1 week after AVR

| No. | LVEDV(ml) | LVESV(ml) | LVEF(%) | GLS(%) | GCS(%) |
| --- | --- | --- | --- | --- | --- |
| 1 | 90 | 53 | 40 | -16 | -13 |
| 2 | 107 | 56 | 45 | -8 | -14 |
| 3 | 86 | 45 | 46 | -10 | -16 |
| 4 | 90 | 47 | 46 | -13 | -14 |
| 5 | 81 | 34 | 56 | -16 | -25 |
| 6 | 110 | 72 | 34 | -6 | -9 |
| 7 | 104 | 57 | 45 | -10 | -15 |
| 8 | 84 | 35 | 56 | -16 | -26 |
| 9 | 87 | 49 | 43 | -13 | -18 |
| 10 | 94 | 68 | 27 | -6 | -6 |
| 11 | 117 | 71 | 39 | -5 | -8 |
| 12 | 84 | 47 | 44 | -14 | -22 |
| 13 | 103 | 68 | 33 | -7 | -10 |
| 14 | 82 | 35 | 56 | -15 | -21 |
| 15 | 83 | 35 | 57 | -16 | -24 |
| 16 | 100 | 62 | 38 | -5 | -7 |
| 17 | 92 | 48 | 47 | -13 | -19 |
| 18 | 88 | 40 | 53 | -20 | -23 |
| 19 | 114 | 72 | 36 | -10 | -7 |
| 20 | 117 | 74 | 36 | -10 | -8 |

Table C The detail information of echocardiographic parameters of AS patients at 1 month after AVR

| No. | LVEDV(ml) | LVESV(ml) | LVEF(%) | GLS(%) | GCS(%) |
| --- | --- | --- | --- | --- | --- |
| 1 | 88 | 50 | 44 | -19 | -16 |
| 2 | 104 | 53 | 49 | -11 | -20 |
| 3 | 84 | 45 | 45 | -16 | -13 |
| 4 | 89 | 45 | 48 | -15 | -18 |
| 5 | 83 | 35 | 57 | -21 | -29 |
| 6 | 107 | 70 | 34 | -6 | -9 |
| 7 | 102 | 56 | 45 | -11 | -17 |
| 8 | 85 | 35 | 58 | -21 | -33 |
| 9 | 87 | 47 | 46 | -17 | -20 |
| 10 | 92 | 61 | 33 | -7 | -5 |
| 11 | 114 | 67 | 41 | -14 | -15 |
| 12 | 85 | 45 | 47 | -16 | -23 |
| 13 | 96 | 62 | 35 | -8 | -11 |
| 14 | 80 | 35 | 56 | -15 | -29 |
| 15 | 80 | 34 | 58 | -16 | -31 |
| 16 | 96 | 59 | 39 | -6 | -8 |
| 17 | 89 | 44 | 50 | -18 | -20 |
| 18 | 82 | 35 | 56 | -24 | -25 |
| 19 | 108 | 68 | 37 | -9 | -9 |
| 20 | 110 | 69 | 37 | -10 | -9 |

Table D The detail information of echocardiographic parameters of AS patients at 3 months after AVR

| No. | LVEDV(ml) | LVESV(ml) | LVEF(%) | GLS(%) | GCS(%) |
| --- | --- | --- | --- | --- | --- |
| 1 | 89 | 40 | 55 | -21 | -26 |
| 2 | 104 | 42 | 59 | -22 | -28 |
| 3 | 86 | 39 | 54 | -19 | -19 |
| 4 | 89 | 40 | 55 | -23 | -24 |
| 5 | 85 | 30 | 64 | -30 | -32 |
| 6 | 107 | 52 | 51 | -14 | -16 |
| 7 | 103 | 45 | 56 | -20 | -24 |
| 8 | 85 | 32 | 62 | -26 | -36 |
| 9 | 87 | 35 | 59 | -23 | -29 |
| 10 | 93 | 47 | 49 | -11 | -15 |
| 11 | 115 | 51 | 54 | -15 | -24 |
| 12 | 86 | 32 | 63 | -26 | -29 |
| 13 | 96 | 36 | 62 | -17 | -24 |
| 14 | 90 | 30 | 67 | -23 | -37 |
| 15 | 82 | 31 | 62 | -22 | -32 |
| 16 | 96 | 47 | 51 | -13 | -18 |
| 17 | 89 | 38 | 57 | -20 | -29 |
| 18 | 86 | 33 | 61 | -21 | -33 |
| 19 | 108 | 45 | 58 | -18 | -22 |
| 20 | 110 | 44 | 60 | -20 | -24 |
