# Supplementary material for: The Early Variation of Left Ventricular Strain after Aortic Valve Replacement by Three-Dimensional Echocardiography
Source: PLoS One. 2015 Oct 16;10(10):e0140469. doi: 10.1371/journal.pone.0140469 (PMC4608801; doi:10.1371/journal.pone.0140469)
Supplement: S2 File — (DOCX) [file pone.0140469.s002.docx]

Table A The detail information of echocardiographic parameters of AI patients before AVR

| No. | LVEDV(ml) | LVESV(ml) | LVEF(%) | GLS(%) | GCS(%) |
| --- | --- | --- | --- | --- | --- |
| 1 | 203 | 128 | 37 | -15 | -16 |
| 2 | 230 | 122 | 47 | -13 | -18 |
| 3 | 137 | 95 | 32 | -7 | -8 |
| 4 | 225 | 142 | 36 | -9 | -10 |
| 5 | 129 | 54 | 58 | -21 | -19 |
| 6 | 120 | 66 | 45 | -13 | -12 |
| 7 | 84 | 35 | 58 | -19 | -23 |
| 8 | 137 | 54 | 61 | -21 | -26 |
| 9 | 245 | 163 | 33 | -9 | -9 |
| 10 | 134 | 74 | 45 | -13 | -15 |
| 11 | 197 | 118 | 40 | -10 | -12 |
| 12 | 185 | 114 | 38 | -11 | -10 |
| 13 | 167 | 93 | 44 | -16 | -18 |
| 14 | 188 | 119 | 37 | -15 | -15 |
| 15 | 183 | 125 | 31 | -8 | -10 |
| 16 | 158 | 107 | 32 | -8 | -8 |

Table B The detail information of echocardiographic parameters of AI patients at 1 week after AVR

| No. | LVEDV(ml) | LVESV(ml) | LVEF(%) | GLS(%) | GCS(%) |
| --- | --- | --- | --- | --- | --- |
| 1 | 139 | 94 | 32 | -5 | -5 |
| 2 | 146 | 97 | 33 | -8 | -6 |
| 3 | 89 | 61 | 31 | -5 | -5 |
| 4 | 165 | 123 | 25 | -4 | -3 |
| 5 | 85 | 39 | 54 | -14 | -15 |
| 6 | 88 | 45 | 49 | -10 | -11 |
| 7 | 81 | 38 | 53 | -15 | -13 |
| 8 | 92 | 43 | 53 | -18 | -16 |
| 9 | 207 | 112 | 46 | -12 | -12 |
| 10 | 104 | 67 | 35 | -7 | -9 |
| 11 | 158 | 91 | 42 | -9 | -9 |
| 12 | 141 | 88 | 37 | -9 | -9 |
| 13 | 129 | 69 | 46 | -12 | -12 |
| 14 | 152 | 95 | 37 | -9 | -12 |
| 15 | 128 | 89 | 30 | -5 | -4 |
| 16 | 110 | 71 | 35 | -7 | -6 |

Table C The detail information of echocardiographic parameters of AI patients at 1 month after AVR

| No. | LVEDV(ml) | LVESV(ml) | LVEF(%) | GLS(%) | GCS(%) |
| --- | --- | --- | --- | --- | --- |
| 1 | 110 | 80 | 27 | -4 | -6 |
| 2 | 120 | 82 | 32 | -7 | -8 |
| 3 | 93 | 47 | 49 | -12 | -14 |
| 4 | 139 | 110 | 21 | -3 | -5 |
| 5 | 86 | 32 | 63 | -14 | -16 |
| 6 | 92 | 35 | 62 | -18 | -16 |
| 7 | 76 | 33 | 56 | -14 | -15 |
| 8 | 88 | 34 | 61 | -16 | -18 |
| 9 | 184 | 97 | 47 | -11 | -12 |
| 10 | 90 | 55 | 39 | -9 | -11 |
| 11 | 133 | 80 | 40 | -9 | -7 |
| 12 | 127 | 75 | 41 | -9 | -11 |
| 13 | 106 | 54 | 49 | -11 | -15 |
| 14 | 130 | 85 | 34 | -9 | -10 |
| 15 | 111 | 76 | 31 | -6 | -6 |
| 16 | 103 | 58 | 43 | -10 | -12 |

Table D The detail information of echocardiographic parameters of AI patients at 3 months after AVR

| No. | LVEDV(ml) | LVESV(ml) | LVEF(%) | GLS(%) | GCS(%) |
| --- | --- | --- | --- | --- | --- |
| 1 | 105 | 55 | 48 | -18 | -21 |
| 2 | 121 | 67 | 44 | -16 | -16 |
| 3 | 88 | 31 | 65 | -30 | -28 |
| 4 | 132 | 74 | 44 | -16 | -17 |
| 5 | 88 | 30 | 66 | -27 | -29 |
| 6 | 91 | 33 | 64 | -28 | -28 |
| 7 | 82 | 32 | 61 | -25 | -25 |
| 8 | 87 | 29 | 67 | -29 | -32 |
| 9 | 165 | 78 | 53 | -22 | -23 |
| 10 | 88 | 36 | 59 | -24 | -25 |
| 11 | 127 | 63 | 50 | -20 | -20 |
| 12 | 121 | 58 | 52 | -21 | -21 |
| 13 | 105 | 46 | 56 | -27 | -24 |
| 14 | 125 | 69 | 45 | -17 | -18 |
| 15 | 108 | 54 | 50 | -19 | -18 |
| 16 | 105 | 43 | 59 | -24 | -27 |
